# Retinal and vitreous metastases from hepatocholangiocarcinoma

**DOI:** 10.1186/s12885-017-3429-8

**Published:** 2017-06-19

**Authors:** Anna Praidou, Sarita Jacob, Luciane Irion, Ramesh Sivaraj, Carl Groenewald, Sarah E. Coupland, Heinrich Heimann

**Affiliations:** 1 Ocular Oncology Service, St Paul’s Eye Unit, Royal Liverpool University Hospital, L7 8XP, Liverpool, UK; 2 Department of Ophthalmology, Heart of England NHS Foundation Trust, Birmingham, UK; 3 Department of Molecular and Clinical Cancer Medicine, Institute of Translational Medicine, University of Liverpool, Liverpool, UK

**Keywords:** Metastasis, Hepatocholangiocarcinoma, Vitreous, Retina, Eye, Metastases

## Abstract

**Background:**

To report a case of metastatic hepatocholangiocarcinoma to the vitreous and retina.

**Case presentation:**

A 70-year-old male, who was recently diagnosed with hepatocholangiocarcinoma, was complaining of floaters in his right eye over the past 5 months and was referred to the Liverpool Ocular Oncology Centre. On presentation, his visual acuity in the right eye was 6/24. Fundus exam revealed a whitish, unilateral, full-thickness retinal lesion at the inferotemporal arcade of his right eye, with vitreous infiltration and subretinal fluid. The patient underwent 25G pars plana vitrectomy with biopsy, resection of the lesion and intravitreal bevacizumab injection. Histopathology testing of the surgical specimens confirmed the diagnosis of metastatic carcinoma to the eye. Two months postoperatively his visual acuity had improved to 6/7.5 and there was no sign of active disease in his right eye, while 9 months postoperatively his visual acuity decreased to 6/9.5 due to developing nuclear sclerotic cataract in his right eye.

**Conclusion:**

The current report presents the first case of a hepatocholangiocarcinoma metastasis to the vitreous and retina.

## Background

Hepatocellularcarcinoma (HCC) and cholangiocellularcarcinoma (CCC) within the same liver, is designated as hepatocholangiocarcinoma (HCC/CCC) [1]. Uveal metastasis is the most common intraocular malignancy, [2] while retinal metastasis is rare [3]. Retinal metastasis from HCC/CCC was not previously reported in the literature. We present the first case of metastatic spread of HCC/CCC to the retina and vitreous.

## Case presentation

A 70-year-old male was referred to Liverpool Ocular Oncology Centre (LOOC), complaining of floaters in his right eye (RE) over the past 5 months. One month before presentation to LOOC, the patient presented to Oncology Department with abdominal pain, lack of appetite and weight loss. The patient was diagnosed by Medical Oncologists with HCC/CCC with multiple liver metastases. The diagnosis of HCC/CCC was made via endoscopic ultrasound (EUS) and fine-needle aspiration (FNA) with 19G needle of the primary tumour which sent for histology and confirmed the diagnosis. Computed tomography (CT) scan confirmed the presence of liver metastases. The staging of the tumour was T3N1M1 and/or stage IV B (liver metastases).

**Fig. 1:**
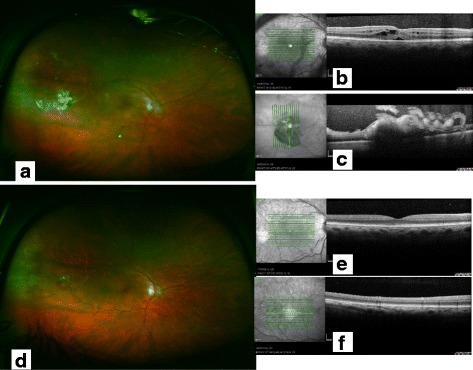
**a** Colour wide angle fundus picture of the right eye of the patient at presentation showed a single whitish elevated lesion at the infero-temporal arcade associated with vitreous infiltration and retinal fluid, **b** Spectral Domain OCT of the right macula of the patient at presentation showing intraretinal and subretinal fluid, **c** Spectral Domain OCT through the lesion at presentation showing full thickness retinal lesion with vitreous involvement **d** Colour wide angle fundus picture of the right eye of the patient two months postoperatively showed clear vitreous, no new retinal lesion and no signs of any active eye disease, **e** Spectral Domain OCT of the right macula of the patient two months postoperatively showing complete regression of the retinal fluid, **f** Spectral Domain OCT through the lesion two months postoperatively showing total anatomical restoration of the retinal layers

**Fig. 2:**
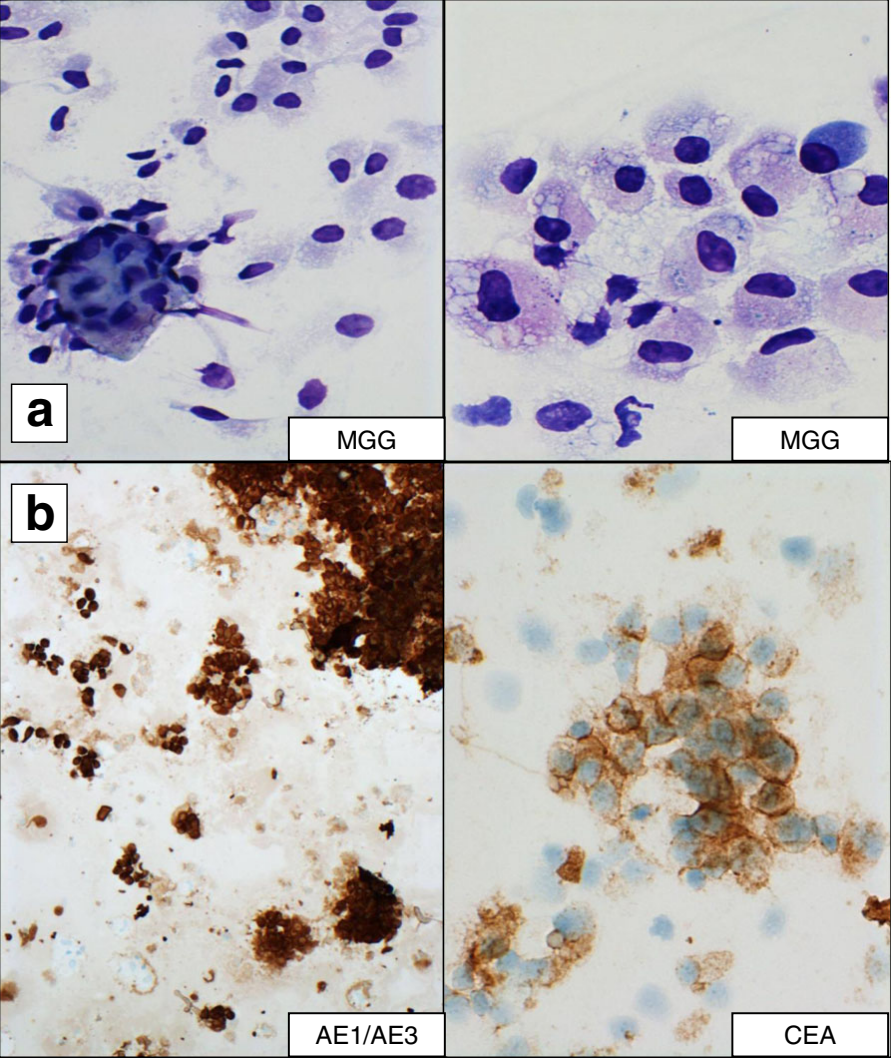
Histopathology examination of retinal and vitreous specimens established the diagnosis of metastatic carcinoma; **a** Cytology preparation stained with May-Grunwald Giemsa stain (MGG) showing groups of atypical epithelioid cells, some of which with cytoplasmic vacuolation; **b** Immunocytochemistry demonstrating cytoplasmic and membranous expression of tumour cells by pan-epithelial marker AE1/AE3 and carcinoembryonic antigen (CEA)

On presentation to LOOC, best corrected visual acuity (BCVA) was 6/24 in RE and 6/4.8 in left eye. Fundus examination of his RE showed a single whitish elevated lesion 3–4 DD in size at the infero-temporal arcade associated with vitreous infiltration and intra- and sub-retinal fluid. (Fig. 1a-c) The patient underwent a 25G pars plana vitrectomy with excisional biopsy of the affected tissue, resection of the lesion, peeling of the epiretinal membrane, air tamponade and intravitreal bevacizumab (anti-VEGF) injection. Four surgical specimens were sent for histopathology test which confirmed the diagnosis of metastatic carcinoma to the vitreous and retina (Fig. 2). The patient was started on cycles of palliative chemotherapy intravenously with gemcitabine 1000 mg/m^2^ on days 1 and 8 along with cisplatin 70 mg/m^2^ on day 1, after intraocular metastatic disease was diagnosed.

One month postoperatively, his BCVA has improved to 6/9.5 in his RE and fundoscopy revealed peripheral retinal haemorrhage and subretinal haemorrhage near the resolving metastatic lesion. Two months postoperatively the BCVA had improved to 6/7.5 and there was no sign of active disease in his RE (Fig. 1d-f). Four months postoperatively fundoscopy showed telengiectatic vessels on thickened retina with no vitreous infiltration. Six months postoperatively there were pigmentary changes at the site of metastatic lesion, the vitreous was clear and there were no new lesions. Nine months postoperatively his BCVA decreased to 6/9.5 due to developing nuclear sclerotic cataract in his RE, while the vitreous was clear, with no reactivation and no new ophthalmic lesions. Metastatic intraocular lesion had stable regression after treatment with vitrectomy, excisional biopsy and systemic chemotherapy for the duration of ocular follow-up.

The patient underwent in total 8 cycles of palliative chemotherapy with gemcitabine along with cisplatin approximately every 4 weeks but unfortunately succumbed to widespread metastatic disease 5 weeks after completion of last chemo cycle and one year after the initial diagnosis of the primary disease.

## Discussion

HCC/CCC has emerged as a distinct subtype; it represents less than 1% of primary liver cancers [4]. There are no clearly defined diagnostic criteria, and no guidelines regarding therapy [5]. Known risk factors include cholelithiasis, cirrhosis and viral hepatitis [4, 5]. HCC/CCC metastasizes through the venous or lymphatic system or along the biliary lumen with a predilection for the liver [4]. Its distant metastases are rare; however, bony metastases were noted in one patient [5]. There is only one case in the literature of a solitary lacrimal gland tumor from metastatic HCC [6].

Metastatic lesions are the most common intraocular malignancies. The majority of intraocular metastases affect the choroid, while the ciliary body, the iris, the retina and the vitreous are less frequently affected. The current case illustrates the first case of a rare site of metastasis to the vitreous and retina for HCC/CCC. Breast and lung cancer most commonly metastasises to the uvea [2, 3]. Patients with ocular metastasis most often present with blurring vision, metamorphopsia, floaters, photopsia, and a visual field defect [2]. Our patient was complaining of floaters in his RE over the past 5 months. Retinal metastases are usually unilateral, appear white, yellow, or brown; are located in the inner retina or full-thickness retina; and have vitreous infiltrates, vitreous hemorrhage, retinal hemorrhage, subretinal fluid, intraretinal exudation [3] and are initially often misdiagnosed as retinitis, hemangioma, choroidal neovascular membrane, or nerve fiber layer infarction [3]. In the present case it was a unilateral white, full-thickness retinal lesion with vitreous infiltration and subretinal fluid, which resembled the clinical picture of a localized retinitis.

As the patient presented with a recently diagnosed systemic malignancy and some primary tumours tend to metastasize to the retina and vitreous, the suspected diagnosis was that of retinal and vitreous metastatic lesion. The differential diagnosis included fungal endophthalmitis an infectious retinitis of unknown origin. The biopsy was performed to establish the suspected diagnosis of an intraocular metastasis against the background of the unusual location of the lesion [2, 3] as the treatment for these entities varies significantly and severe and permanent visual loss was to be expected in case of progressive, untreated disease. Our patient was diagnosed with a metastatic HCC/CCC to the vitreous and retina which was confirmed by biopsy. Treatment of ocular metastasis includes radiotherapy, resection with/out intravitreal anti-VEGF injection, enucleation, observation along with/or systemic chemotherapy or biologic therapy [2, 3].

## Conclusion

In conclusion, the current report presents the first case of unusual HCC/CCC metastasis to the vitreous and retina. A retinal and vitreous biopsy was required to establish the diagnosis, while the therapeutic options for this case included local treatment for the intraocular metastasis and systemic chemotherapy for the primary lesion and its metastatic disease.

## Abbreviations

BCVA: Best corrected visual acuity
CT: Computed tomography
EUS: Endoscopic ultrasound
FNA: Fine-needle aspiration
HCC/CCC: Hepatocholangiocarcinoma
LOOC: Liverpool Ocular Oncology Centre
RE: Right eye
